# GERIATRIC RENAL TRANSPLANT CO-MANAGEMENT: EVALUATION OF FRAIL OLDER ADULTS FOR TRANSPLANT ELIGIBILITY

**DOI:** 10.1093/geroni/igad104.2930

**Published:** 2023-12-21

**Authors:** Jared Doan, Stephanie Chow, Abigail Baim-Lance, Moritz Blum, Susan Lerner, Greta Rosen, William Hung, Fred Ko

**Affiliations:** Icahn School of Medicine at Mount Sinai, New York City, New York, United States; Icahn School of Medicine at Mount Sinai, New York City, New York, United States; Veterans Health Administration, Bronx, New York, United States; Icahn School of Medicine at Mount Sinai, New York City, New York, United States; Icahn School of Medicine at Mount Sinai, New York City, New York, United States; Icahn School of Medicine at Mount Sinai, New York City, New York, United States; Icahn School of Medicine at Mount Sinai, New York City, New York, United States; Icahn School of Medicine at Mount Sinai, New York City, New York, United States

## Abstract

Older transplant patients have higher rates of adverse post-transplant outcomes. The Renal Transplant Co-Management (RCOM) program is a collaboration of surgeons, geriatricians, and social workers to medically and psychosocially optimize older renal transplant candidates by providing comprehensive geriatric-centered vulnerability assessments with corresponding medical and psychosocial interventions. The RCOM assessment aids the surgical team in deciding patient transplant eligibility, and greater clarity on its role may be clinically valuable. Fifty-five patients from a single metropolitan hospital were evaluated during the study period (1/1/22 to 6/16/22). Assessments included frailty (Clinical Frailty Scale/CFS), functional status (Karnofsky Score, Katz Index, Lawton-Brody Scale) and cognition (Montreal Cognitive Assessment/MoCA) – factors that the renal transplant team considers in listing a patient for surgery (i.e., listed/transplanted) or not (i.e., ineligible/removed from the list). Twenty patients (36.4%) were female. Mean age of ineligible/removed (n=10) and listed/transplanted (n=45) patients was 74.7 (4.8) and 72.5 (3.8), respectively, with no differences in age, gender, race, education, or health insurance between groups. Patients who were listed/transplanted had a higher Lawton-Brody instrumental activities of daily living (IADL) score than those not listed (p=0.025, t-test). CFS (p=0.118), Karnofsky (p=0.09), Katz (p=0.074), and MoCA (p=0.094) were similar but approached statistical significance despite a small sample size. Functional status as measured by IADL may be a significant factor considered in determining the transplant eligibility of older adults. Ongoing study and a larger sample size may provide greater clarity on the physical and cognitive functions that impact eligibility for transplant listing in frail older adults.

